# Improving international medical students’ communication skills in psychosomatic care through a communication training seminar: a natural language processing-based analysis

**DOI:** 10.1186/s12909-026-09890-5

**Published:** 2026-07-11

**Authors:** Julia Sgrott, Christoph Nikendei, Ede Nagy, Aleksei Smirnov, Josefina Arias Alvarado, Hans-Christoph Friederich, Ivo Dönnhoff

**Affiliations:** 1 Department for General Internal Medicine, Psychosomatics and Psychotherapy, Heidelberg University Hospital, Thibautstraße 4, Heidelberg, 69115 Germany; 2 Heidelberg’s Tutorial for International Medical Students (HeiTiMed), University of Heidelberg, Heidelberg, Germany; 3 DZPG – German Center for Mental Health Mannheim-Heidelberg-Ulm, Heidelberg University Hospital, Heidelberg, Germany

**Keywords:** International students, Natural language processing, Communication training, Patient-centered communication

## Abstract

**Background:**

International students face numerous challenges in both their academic and social lives when relocating to a new country for their university studies. The foreign language can have an impact on international medical students’ performance in patient encounters. Particularly in the context of psychosocial medicine, a deficiency in communication skills can negatively impact the doctor-patient-relationship. This is the first study that uses natural language processing (NLP) as well as traditional rating scales to assess the improvement of international students’ communication skills after attending a communication training seminar.

**Methods:**

N = forty-two international students participated in the study, which followed a pre-post-design. Diagnostic interviews with standardized patients (SP) were videotaped before and after the three-day training seminar, which was comprised of short lectures and communication trainings with SPs regarding the topic of psychosomatic medicine. Transcripts of diagnostic interviews were analyzed with NLP. Videotaped clinical encounters were assessed by three raters with binary and global rating instruments. For the comparison between pre- and post-assessment of all NLP communication parameters and rating results, two-way ANOVAs and Wilcoxon tests were calculated, respectively.

**Results:**

NLP communication parameter results showed a decrease in talk-turns, interruptions, number of questions and talking over, and an increase in talk-turn-length in post-assessment compared to pre-assessment. There were no significant pre-post changes observed in binary checklist results. Significant pre-post changes were observed in global rating score and ratings from global rating domains ‘interview structure’ and ‘verbal expression’.

**Conclusion:**

International students significantly improved their communication style in psychosomatic medicine towards a more patient-centered approach. Changes in NLP communication parameters and improved interview style and verbal expression suggest that international students may have listened to patients more carefully. Furthermore, NLP has shown to be a viable tool to evaluate communication parameters in a doctor-patient-relationship and assess the effectiveness of communication training for international students.

**Supplementary Information:**

The online version contains supplementary material available at 10.1186/s12909-026-09890-5.

## Background

International students are defined as students who achieved their university entry qualification in their home country and moved abroad for university education [1]. Germany has consistently been an attractive country for international medical students, who represent around 13% of the total of medical students in the country [1, 2]. Several authors [3, 4] have previously discussed the challenges that international medical students face in the pursuit of a university degree. These challenges include limited social and financial support [3, 4], personal distress [5], language barriers [6, 7], and cultural barriers [8]. These may contribute to a lower academic performance in written, oral, and practical examinations [9–12]. In the field of psychosocial medicine, Huhn et al. showed that international students scored significantly worse in the Objective Structured Clinical Examination (OSCE), compared to German students in scenarios with psychiatric and psychosomatic standardized cases [13].

As part of psychosocial medicine, the interdisciplinary field of psychosomatics has emerged in the last few decades [14]. Psychosomatics investigates the role of psychosocial factors and their interaction with biological factors in the course and outcome of all types of diseases [15]. Moreover, psychosomatics integrates psychotherapeutic methods in diverse clinical fields, promoting a deeper comprehension of patients’ vulnerability to a disease and an improvement in patients’ quality of life [15, 16]. Specifically in this field, acquiring a set of communication skills to build a doctor-patient-relationship is of utmost importance [17]. Challenges, such as understanding patients’ narrated biography and the complexity of their needs and affects, can be more easily overcome with a patient-centered communication [17]. Patient-centered communication encompasses actively listening to patients, avoiding interruptions and talking over, and making use of non-verbal communication [18]. However, the establishment of this style of communication by international medical students, especially in this medical field, can likely be affected by cultural facets and language difficulties as well as by a lack of familiarity with psychosomatic concepts [19, 20]. Furthermore, a lack of confidence when speaking the foreign language [21] could further contribute to a weaker patient-centered communication and, thus, doctor-patient-relationship. The aforementioned difficulties can impact international students’ ability to recognize important verbal and non-verbal cues, which are key to a deeper understanding of patients’ feelings and subjective illness theory.

There are several tools that can be used to assess medical students’ communication skills [22]. ‘Natural language processing’ (NLP) has been developing in medical education as an evaluation tool, which has been, so far, mostly employed to evaluate students’ communicative performance in communication training with the goal of improving medical educators’ feedback [23, 24]. NLP analyzes human’s natural spoken or written language, using statistical methods [25]. With the increase in computational power, NLP has been growing in popularity [26], including in the medical field [27]. However, NLP has not yet been applied in the medical education field as a method to analyze international students’ communicative styles and speech dynamics in a doctor-patient-relationship. Applying NLP as an analysis tool of international students’ communication skills could provide more insights regarding international students’ communication difficulties in the doctor-patient-relationship. An enhanced understanding of these difficulties could lead to further improvements in communication training for international medical students. Through traditional ratings, communication training has already proven to be a valuable tool to support the development of international students’ communication skills [28, 29]. However, thus far, this has only been applied outside of the field of psychosomatics.

The presented study was designed as a pilot study with the aim of investigating international medical students’ communication development through communication training. A three-day peer-led communication training seminar was developed exclusively for international medical students. During training, international students actively practiced diagnostic interviews with psychosomatic standardized patients. We had the following two primary expectations:

1. Changes in NLP communication parameters and rating results would reflect a shift of international students’ communication style towards a more patient-centered communication.
  - The NLP analysis would show that international students (i) interrupt less and (ii) talk over patients less, thereby allowing patients to have longer talk-turns and so, demonstrating an improved active listening.
  - By being more assertive in their communication, international students (iii) would use fewer filler words such as ‘um’, which usually signal uncertainty and nervousness [30].
  - Regarding rating results, we expected that by (iv) listening more and asking more targeted, diagnostically relevant questions, international students would (v) be perceived as more understanding and confident and, therefore, (vi) receive higher ratings for patient-centered communication.
2. The NLP method would show to be a viable tool to evaluate communication parameters in the doctor-patient-relationship and thus, enrich the assessment of communication training alongside traditional rating methods.

## Methods

### Study design

This prospective pilot study was conducted in a pre-post-assessment design. A three-day peer-led training seminar provided international medical students with information on mental illnesses through short lectures and training of diagnostic interviews with psychosomatic standardized patients (SP). Short lectures and diagnostic interviews were conducted in German. The training seminar was organized by the Heidelberg’s Tutorial for International Medical Students (HeiTiMed) [5]. All videotaped diagnostic interviews were manually transcribed and later analyzed with NLP. The possible changes in international students’ communication skills were assessed with a binary checklist and a global rating scale [31, 32].

### Participants

For the purposes of this study, international students are defined as students who obtained their university entry qualification abroad and moved to Germany in pursuit of higher education. International medical students enrolled at Heidelberg University in their second study year or higher were invited to participate in the training seminar. The study was conducted once in 2020, 2021, 2023 and 2024. In total *n* = 42 international students participated in this study.

### Training design

**Fig. 1:**
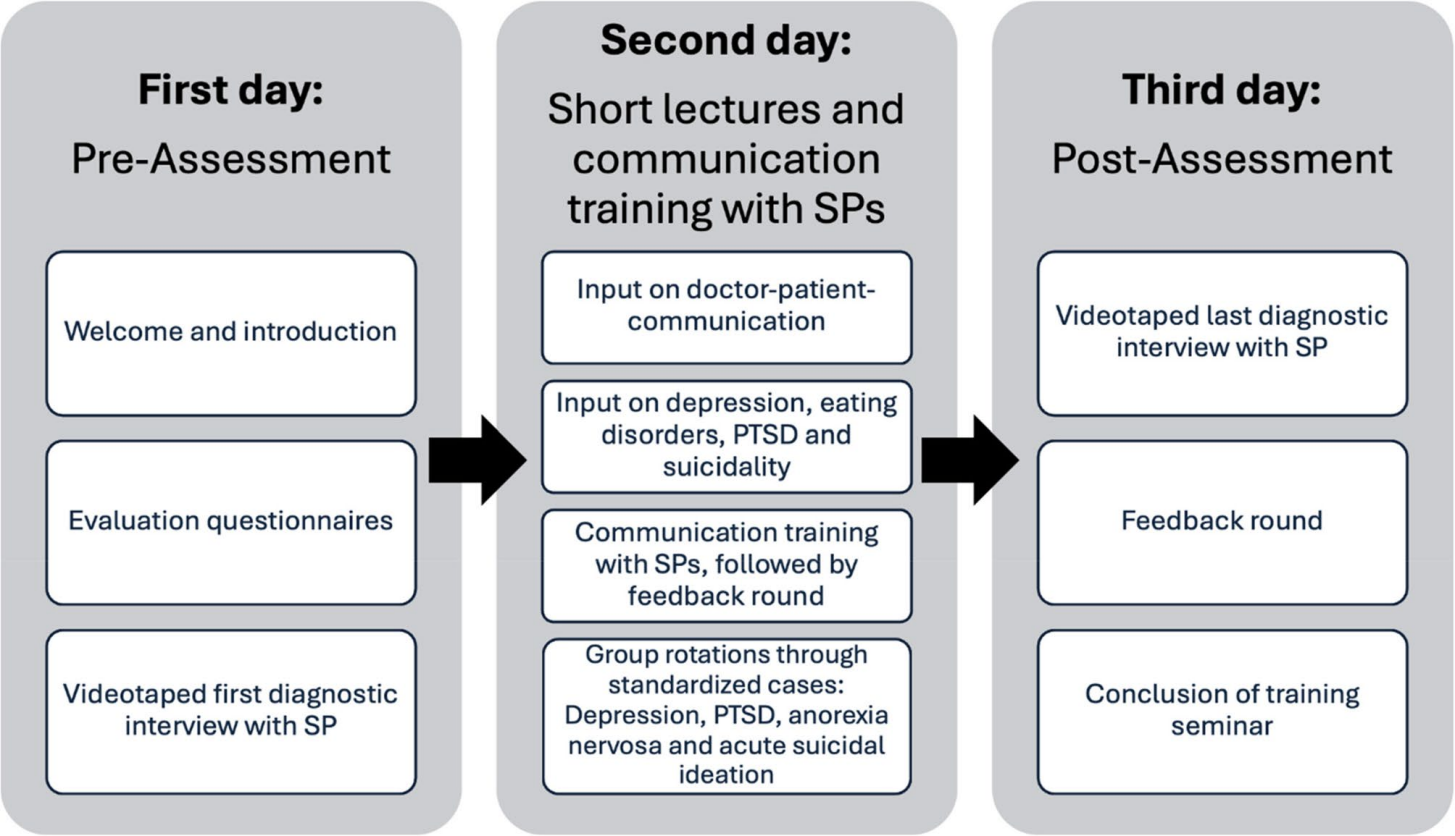
Training seminar design

The training design can be seen in Fig. 1. The three-day training seminar was offered once a year during the winter term. On the first day of the seminar, international students took part in pre-assessment. On the second day, short lectures were given by tutors. Tutors provided input on symptoms, diagnostic guidelines, and therapeutic procedures for depression, posttraumatic stress disorder (PTSD), anorexia nervosa, bulimia nervosa and acute suicidal ideation. Furthermore, the short lectures set focus on communication skills and communication theories. For the training session, international students were randomly divided into groups of six students. One student conducted a ten-minute long diagnostic interview with an SP while the others observed. International students were not provided with an interview guide. International students were instructed to follow the logical flow of conversation established by the patient while assessing the possible diagnosis. The diagnostic interview was followed by a feedback round, in which the interview-conducting student, SP, the group, and the tutor provided their feedback, in this respective order. During the training session, each student had the opportunity to carry out at least two diagnostic interviews. On the last day, international students took part in post-assessment and evaluated the training seminar.

### Tutors

The seminar was organized by the Heidelberg’s Tutorial for International Medical Students (HeiTiMed). HeiTiMed focuses on supporting international students academically and socially, by offering weekly revision tutorials for international students in their first and second year and organizing social gatherings [5]. HeiTiMed tutors are international students themselves, who are in the third or higher year of their medical studies. Seminar tutors were HeiTiMed tutors who have had previous communication training throughout their university studies. Tutors were responsible for preparing presentation materials, carrying out short lectures and providing feedback on diagnostic interviews. Furthermore, the seminar was supervised by two licensed psychotherapists with at least five years of clinical experience, who provided their input.

### Psychosomatic standardized patients (SP)

SPs were actors contracted by Heidelberg’s Medical Faculty [33]. SPs participating in the training seminar had previous experience with other medical communication trainings. Six months prior to the seminar, SPs received a description of their role. Detailed instructions on patients’ appearance, biography, current symptoms, living situation and personal relationships were provided. Interview directions focused on giving SPs an orientation on how interviews should progress based on international students’ exploration of patients’ symptoms, personal life and concerns. SPs portrayed the following disorders: depression, PTSD, anorexia nervosa and acute suicidal ideation.

### Procedure and assessment

On the first day, international students took part in pre-assessment. International students filled out a short questionnaire with demographic questions, such as gender, country of origin, semester, age and previous medical experience. Next, international students performed a ten-minute diagnostic interview with an SP, which was videotaped. International students did not receive any input from tutors prior to the first interview. There were two possible standardized cases for the assessment interview: ‘depression’ or ‘PTSD’. International students were randomly assigned one of these cases. Then, international students participated in training as described above. On the last day, international students carried out their last diagnostic interview with an SP as post-assessment, which was also videotaped. International students who randomly received ‘depression’ as their pre-assessment case were assigned ‘PTSD’ for post-assessment, while those who randomly received ‘PTSD’ in pre-assessment were assigned ‘depression’ as their post-assessment case. ‘Depression’ and ‘PTSD’ cases portrayed in pre- and post-assessment were the same cases practiced in training. Readers interested in the technical setup for pre- and post-assessment are referred to supplement A.

### Transcription and development of NLP communication parameters

To analyze the spoken language from international students with the method of NLP, all 78 videos were manually transcribed by two HeiTiMed tutors (the first and fourth author of this manuscript). Transcription rules were developed within the research group, ensuring a standardization of transcription in all research projects. A few examples of these transcription rules include transcribing all words, fillers, such as ‘um’ and ‘mhm’, and partially spoken words. Certain speech interactions such as interruptions and talking over were transcribed and marked with special characters. The fillers ‘um’ und ‘mhm’ and the marked speech interactions were the NLP communication parameters selected for analysis. The fillers ‘um’ and ‘mhm’ were analyzed separately, as each filler has their own attributed meaning: while ‘um’ is perceived as a sign of uncertainty and nervousness, ‘mhm’ is mostly used to indicate active listening [30, 34]. Table 1 provides an explanation for each analyzed NLP communication parameter and the special character used to mark it in transcripts. The interested reader is referred to DOI 10.11588/DATA/2ETDYP. There, we have uploaded our transcription rules in detail. After the transcription phase was finished, all transcripts were revised by the first author while rewatching the video recordings and therefore, ensuring the uniform application of transcription rules.

**Table 1:** NLP communication parameters

Parameter	Definition	Special character
Talk-turn	Uninterrupted segment of speech delivered by one speaker	T = student’s talk-turn P = patient’s talk-turn
Talk-turn-length	Number of words in a single talk-turn	
Filler sounds	Small vocalizations in speech to pause, think, or reassure	‘Ähm’ = ‘um’ ‘Mhm’
Questions	Requesting information, clarify understanding	?
Interruptions	A speaker begins to talk while the previous speaker is still talking, causing a disruption of the talk-turn. The previous speaker stops speaking after the interruption	A slash '/' signaled the interruption of the talk-turn from the previous speaker P: xxx / T: xxx
Talking over	A speaker begins to talk while the previous speaker is still talking. In other words, both speakers talk at the same time	Overlapped speech segments from both speakers were put between double slashes: P: xxx // xxx // T: // xxx // xxx

### Rating instruments

A binary checklist and a global rating scale were utilized to evaluate the improvement of international students in communication skills and in relationship building. The binary checklist focused on evaluating international students’ abilities in assessing patients’ history and relationship building. The global rating scale concentrated on the evaluation of communication skills. A combination of global and binary ratings has been applied to promote a more precise and effective assessment method of communication skills in a doctor-patient-relationship [35]. Binary items capture single aspects of the history-taking process, whereas global rating scales encompass aspects of clinical competence, such as empathy and communication, which tend to be overlooked by binary rating scales [36]. The binary checklist (performed/not performed) was based on the checklists employed in the psychosocial medicine OSCE at Heidelberg University [13] and on the instructions provided by Nikendei and Jünger [31]. The binary checklist was divided in the following domains: ‘relationship-building’ (5 items), ‘conversational skills’ (5 items), ‘anamnesis’ (5 items), and ‘illness-specific symptoms’ (15 items). Each item awarded one point when performed. The domain ‘illness-specific symptoms’ was divided in two specific subdomains: ‘depression symptoms’ and ‘PTSD symptoms’. For each subdomain, 15 symptoms were listed as items. International students received one point per item (maximum of 10 points) when a symptom was mentioned either by SPs or international students in conversation. Only the subdomain associated with the respective case was rated. All points were summarized to a total score of a maximum of 25 points. As a validated global rating instrument, the Berliner Global Rating Scale (BGR) was employed to assess communication skills [32, 37]. It contains the domains ‘empathy’, ‘interview structure’, ‘verbal expression’, and ‘non-verbal expression’. Raters attributed a grade for each domain from a numeric ordinal scale from 1 to 5, meaning: 1 = excellent, 2 = good, 3 = satisfactory, 4 = sufficient, 5 = poor. A mean of the scores from all domains referred to an overall global rating score. The interested reader is referred to DOI 10.11588/DATA/2ETDYP. There, we have uploaded all rating instruments in their entirety.

Prior to the start of the rating process, three HeiTiMed tutors (the first, fourth and fifth authors), who were recruited as raters, participated in a rater training. The interested reader is referred to supplement B, in which the rater training and the rating process are explained in detail.

### Ethics

This study was conducted in accordance with the principles of the Declaration of Helsinki (64th WMA General Assembly, Fortaleza, Brazil, October 2013). The participation in the study was entirely voluntary. All participants were fully informed about the study’s purpose and assured of anonymity of their data. Written informed consent was provided by all participants. The study received ethical approval from the Ethics Committee of the University of Heidelberg (No. S-535/2016).

### A priori power analysis

An a priori power analysis was conducted using G*Power 3 [38]. For the analysis of NLP communication parameters with ANOVAs, assuming an effect size of f = 0.25 and a significance level of α = 0.05 for within-between interactions, a total sample size of 34 participants was required to achieve a power of β = 0.80. For the analysis of rating results with Wilcoxon tests, a total sample size of 35 participants was needed to achieve a power of β = 0.80, considering an effect size d = 0.5 and a significance level of α = 0.05. Over a four-year period, 42 international students participated in this study, with 36 participants taking part in both pre- and post-assessments.

### Inter-rater reliability (IRR)

Inter-rater reliability was assessed with Gwet’s agreement coefficient 2 (AC2) [39], which is less susceptible to paradoxes than other IRR methods. AC2 values are interpreted as in other IRR methods [40].

### Data analysis

All data analysis was carried out with the statistic software R [41].

#### NLP communication parameters

For a definition of the following analyzed NLP communication parameters, we refer the reader to Table 1. The average and total amount of talk-turns, questions, interruptions, and talking over was calculated for both speakers (student and SP) in pre- and post-assessment. The frequency of fillers, such as ‘um’ and ‘mhm’ and the average talk-turn-length, were also analyzed for both speakers and pre- and post-assessments. To compare results between pre- and post-assessment and between speakers, two-way ANOVAs were performed for each parameter. ANOVA-prerequisites were previously checked. Outliers were included in the analysis. The normal distribution was assessed with QQ-Plots. The homogeneity of variances was checked with the Levene test. All communication parameters were in accordance with ANOVA-prerequisites.

Furthermore, following a reviewer’s suggestion, we conducted a controlled statistical analysis to investigate possible differences in the use of NLP communication parameters between the student groups built through the randomization of assessment cases (group 1 = pre-assessment ‘depression’ + post-assessment ‘PTSD’ vs. group 2 = pre-assessment ‘PTSD’ + post-assessment ‘depression’). While the overall results presented in this manuscript remained consistent, an interesting pattern emerged. Differences in communication patterns between the assessment cases (‘depression’ / ‘PTSD’) were substantial and appeared to overshadow improvements in students’ communication performance when not accounted for. The interested reader is referred to supplement C, in which we presented the results of this controlled statistical analysis. Under the DOI 10.11588/DATA/2ETDYP we have uploaded our analysis code.

#### Rating data

For each video, we first calculated the mean score across raters for the binary checklist domains and total score, as well as for the global rating domains and global rating score. Afterwards, the distribution of these mean scores was analyzed. As mean scores were not normally distributed, we opted for the non-parametric Wilcoxon test. Several Wilcoxon tests were calculated to compare the pre- and post-assessment results of the mean scores for each binary checklist and global rating scale domain and scores. Because the Wilcoxon test is a paired test, the results of international students who did not take part in one of the assessments were not taken into consideration in the test statistic.

### Transparency and openness

Our seminar materials, data frame with rating results, rating instruments, transcription rules, and R markdowns with statistical analysis codes are publicly available under the DOI 10.11588/DATA/2ETDYP.

## Results

### Sample description

All study participants (*n* = 42) matched the study definition of international student. Therefore, all participants were taken into consideration in the analysis. However, five participants only participated in pre-assessment and one participant only in post-assessment. The latter participant also did not provide us with their demographic data. All other participants (*n* = 36, 85.7%) took part in both pre- and post-assessment. The mean age of participants was 22.0 years (SD = 1.6 years). 63.4% of our participants were female (f = 26, m = 15). Most participants (82.8%) were in their 3rd year of medical education or higher. The sample included 28 participants from Europe, 8 from Asia, 3 from Africa, and 2 from the Middle East. In the end of the data collection phase, 78 videos of pre- and post-assessments were obtained.

### NLP analysis results

As shown in Tables 2 and 3, there were significantly less talk-turns in post-assessment compared to pre-assessment. The talk-turn-length is significantly longer for both SPs and international students in post-assessment. The talk-turn-length of SPs is significantly longer in both pre- and post-assessment when compared to international students’ talk-turn-length. Furthermore, the use of the filler ‘um’ by international students decreased significantly. There is a significant difference in the usage of the filler ‘um’ between speakers, as international students make a more frequent use of this filler in comparison to SPs. The use of the filler ‘mhm’ did not change between pre- and post-assessments. Regarding the number of questions, results show a significant interaction effect between session and speaker. International students asked more questions compared to SPs in pre- and post-assessment. However, the number of questions asked by international students reduced in post-assessment. Both SPs and international students were interrupted significantly less in post-assessment, as shown by the mean of interruptions of patients and of international students, respectively. Furthermore, international students talk significantly less over SPs in post-assessment, the same way SPs talk less over international students.

**Table 2:** ANOVA and post-hoc power analysis results for NLP communication parameters

NLP communication parameters	Effect	DF1	DF2	F	*p*	η^2^	Post-hoc power β
Talk-turn							
	Speaker	1	70	0.07	0.93	< 0.01	0.05
	Session	1	70	13.05	< 0.01	0.06	0.82
	Speaker : session	1	70	0.007	0.98	< 0.01	0.05
Talk-turn-length							
	Speaker	1	70	18.31	< 0.01	0.18	0.87
	Session	1	70	10.01	0.002	0.03	0.48
	Speaker : session	1	70	2.35	0.13	0.01	0.15
Filler ‘um’							
	Speaker	1	70	40.69	< 0.01	0.34	1.00
	Session	1	70	6.10	0.016	0.01	0.22
	Speaker : session	1	70	5.47	0.022	0.01	0.20
Filler ‘mhm’							
	Speaker	1	70	48.94	< 0.01	0.32	0.99
	Session	1	70	0.18	0.67	< 0.01	0.06
	Speaker : session	1	70	0.10	0.75	< 0.01	0.06
Questions							
	Speaker	1	70	341.6	< 0.01	0.72	1.00
	Session	1	70	2.53	0.12	0.02	0.34
	Speaker : session	1	70	5.37	0.02	0.04	0.60
Interruptions							
	Speaker	1	70	0.02	0.88	< 0.01	0.05
	Session	1	70	13.0	< 0.01	0.06	0.86
	Speaker : session	1	70	< 0.01	0.98	< 0.01	0.05
Talking over							
	Speaker	1	70	5.5	0.02	0.04	0.30
	Session	1	70	4.96	0.03	0.03	0.51
	Speaker : session	1	70	0.10	0.75	< 0.01	0.06

**Table 3:** Mean$\:\pm\:$standard deviation results for NLP communication parameters

NLP communication parameters	Assessment	Mean$\:\pm\:$SD	
		Student	Patient
Talk-turn			
	Pre	59.68$\:\pm\:$44.06	60.08$\:\pm\:$43.95
	Post	48.34$\:\pm\:$35.70	48.76$\:\pm\:$35.40
Talk-turn-length			
	Pre	9.85$\:\pm\:$13.01	13.53$\:\pm\:$16.99
	Post	10.86$\:\pm\:$14.43	16.22$\:\pm\:$22.87
Filler ‘um’			
	Pre	0.07$\:\pm\:$0.06	0.01$\:\pm\:$0.01
	Post	0.06$\:\pm\:$0.04	0.01$\:\pm\:$0.01
Filler ‘mhm’			
	Pre	0.03$\:\pm\:$0.02	0.01$\:\pm\:$0.01
	Post	0.03$\:\pm\:$0.02	0.01$\:\pm\:$0.01
Questions			
	Pre	24.76$\:\pm\:$7.64	4.12$\:\pm\:$3.98
	Post	20.60$\:\pm\:$6.07	4.68$\:\pm\:$3.56
Interruptions			
	Pre	3.51$\:\pm\:$3.65	3.56$\:\pm\:$4.21
	Post	2.19$\:\pm\:$2.56	2.11$\:\pm\:$2.60
Talking over			
	Pre	11.49$\:\pm\:$10.97	7.49$\:\pm\:$7.55
	Post	8.70$\:\pm\:$8.06	5.68$\:\pm\:$6.74

### IRR results

As shown in Table 4, in global rating score and domains, except in the domain ‘verbal expression’, raters showed a moderate agreement. In the ‘verbal expression’ domain, raters showed a substantial agreement. In the binary checklist rating, raters showed a moderate agreement in domains ‘relationship’ and ‘conversational skills’. In ‘anamnesis’ and ‘total score’, the agreement was substantial. In ‘illness-specific symptoms’, raters agreed almost perfectly.

**Table 4:** Inter-rater reliability based on Gwet’s AC2 method

Binary checklist			
Domain	AC Value	Confidence interval	Weights
Relationship	0.56	(0.31,0.82)	Linear
Conversational skills	0.51	(0.23,0.79)	Linear
Anamnesis	0.72	(0.60,0.85)	Linear
Illness-specific symptoms - Depression	0.88	(0.82,0.94)	Linear
Illness-specific symptoms - PTSD	0.87	(0.79,0.94)	Linear
Total score	0.75	(0.48,1)	Linear

Global rating scale			
Domain	AC Value	Confidence interval	Weights
Empathy	0.54	(0.46,0.63)	Linear
Structure	0.51	(0.37,0.66)	Linear
Verbal expression	0.63	(0.39,0.88)	Linear
Non-verbal expression	0.57	(0.40,0.74)	Linear
Global rating score	0.53	(0.39,0.66)	Linear

### Binary checklist results

As seen in Table 5, Wilcoxon test results showed no significant difference in binary rating results between pre- and post-assessment. This applied for total score and single domains.

**Table 5:** Binary checklist and global rating scale Wilcoxon test results, meanstandard deviation and post-hoc power analysis

Binary checklist							
Domain	Pre: Mean$\:\pm\:$SD	Post: Mean$\:\pm\:$SD	V	r	p	d	Post-hoc power β
Relationship building	3.9$\:\pm\:$0.91	3.95$\:\pm\:$0.94	68.5	0.13	0.43	0.14	0.13
Conversational skills	2.83$\:\pm\:$1.02	3.08$\:\pm\:$0.89	88.5	0.26	0.12	0.27	0.34
Anamnesis	4.24$\:\pm\:$0.66	3.86$\:\pm\:$0.98	210.5	0.30	0.07	0.33	0.47
Symptoms* - Depression	4.20$\:\pm\:$4.01	3.57$\:\pm\:$3.98	333.5	0.05	0.77	0.08	0.07
Symptoms* - PTSD	3.46$\:\pm\:$4.06	4.27$\:\pm\:$4.17	254.5	0.08	0.65	0.08	0.07
Total score	18.59$\:\pm\:$2.68	18.65$\:\pm\:$2.37	238.5	0.08	0.64	0.05	0.06

Global rating scale							
Domain	Pre: Mean$\:\pm\:$SD	Post: Mean$\:\pm\:$SD	V	r	p	d	Post-hoc power β
Empathy	2.05$\:\pm\:$0.89	1.95$\:\pm\:$0.74	158	0.18	0.28	0.17	0.16
Interview structure	1.78$\:\pm\:$0.88	1.46$\:\pm\:$0.77	119	0.34	0.04	0.38	0.58
Verbal expression	1.93$\:\pm\:$1.06	1.73$\:\pm\:$0.80	160	0.38	0.02	0.41	0.65
Non-verbal expression	1.39$\:\pm\:$0.63	1.51$\:\pm\:$0.61	56	0.11	0.51	0.11	0.10
Global rating score	1.79$\:\pm\:$0.74	1.67$\:\pm\:$0.50	310.5	0.34	0.04	0.33	0.47

V = Wilcoxon test statistic, p = *p*-value; r = Pearson correlation, d = Cohen’s d

*Domain Illness-specific symptoms

### Global rating scale results

In Table 5, Wilcoxon test results for the domains ‘interview structure’, ‘verbal expression’, and ‘global rating score’ showed a significant increase in rating results from pre- to post-assessment. The rating results for the domains ‘empathy’ and ‘non-verbal expression’ showed no significant differences between pre- and post-assessment.

### Post-hoc power analysis

The results of the post-hoc power analysis for NLP communication parameters are presented in Table 2, while the post-hoc analysis results for the binary checklist and the global rating scale are shown in Table 5. Considerable variability in statistical power was observed across NLP communication parameters and individual domains of both binary and global rating instruments.

## Discussion

To our knowledge, there are no studies that have investigated the impacts of communication training for international students in psychosomatics using the method of NLP. Our results indicate a significant improvement in international students’ communication patterns from pre- to post-assessment, as reflected in the analysis of NLP communication parameters. There are significantly fewer talk-turns between international students and SPs, whereas the talk-turn-length is longer for both speakers. This is consistent with our findings of a significant decrease in interruptions and talking over in post-assessment. International students significantly decreased the use of the filler ‘um’ in post-assessment. Furthermore, international students asked less questions in post-assessment. Binary checklist results did not show a significant difference between pre- and post-assessment. There were significant pre-post changes in the global rating score and in the global rating domains ‘verbal expression’ and ‘interview structure’.

With an increased talk-turn-length, international students and, in particular, patients might have delivered more information. The decrease in talk-turn switches between speakers, interruptions and talking over in post-assessment might have led patients to have an enhanced feeling of being listened to and, hence, might have let them feel more comfortable with speaking freely. Therefore, international students might have reduced the necessity of actively asking for information, as demonstrated by the decrease in questions in post-assessment. Therefore, it may be assumed that international students distanced themselves from a checklist-based interview style, which is characterized by one-sidedly asking questions and focusing exhaustively on symptoms and history-taking [42]. Now, possibly following the logical flow of conversation established by the patient, international students might have been able to avoid constantly interrupting the conversation flow with questions, interruptions and talking over, so that the diagnostic interview might have been generally smoother. This change in interview style could be why the global rating score as well as the global rating domain ‘interview structure’ were evaluated significantly better in post-assessment.

Furthermore, the improvement in NLP communication parameters may reflect the adaptation of international students’ communication style towards a more patient-centered style. The literature describes that a patient-centered doctor-patient-relationship is based on patient-centered-communication, in which physicians actively listen to patients, avoid interruptions, make use of non-verbal communication and address patients’ verbal and non-verbal cues [18, 43]. Therefore, our NLP analysis shows that international students might gradually align to this communication style and thus, partially to a more patient-centered doctor-patient-relationship. Furthermore, the consistent use of the filler ‘mhm’ from pre- to post-assessment may suggest that international students use it as a marker of active listening [34].

The relevance of our findings grows when the language barrier experienced by international students is taken into consideration. Since language barriers can directly impact international students’ ability to establish a doctor-patient-relationship, enhancing their communication skills might also facilitate the building of this relationship, potentially leading to improved patient health outcomes [44].

Moreover, the decrease in usage of the filler ‘um’ by international students may be the result of developing an increased sense of confidence in their communication skills. Fillers, such as ‘um’, could be interpreted as a sign of nervousness and insecurity, related to a lack of preparation [30]. When used excessively, it can impair listeners’ understanding of the speaker’s message [30]. With short lectures and practical training with SPs, international students might have obtained more knowledge about important aspects of patients’ illnesses and more experience with communication skills. Hence, international students could have felt more prepared and confident in performing the diagnostic interview. As previously described by Roberston et al., confidence is key to international students’ expression in the foreign language [21]. Thus, increasing international students’ confidence might have led to a significant improvement of their verbal expression, as shown by global rating scale results. In post-assessment, international students could have communicated more clearly by employing more appropriate and confident use of voice intonation, volume, modulation and speaking pace [45]. However, there were no significant improvements in the ratings of the domain ‘conversational skills’ from the binary checklist, which encompasses the aspects of answering patients’ questions, ensuring patients’ understanding, mirroring important conversation topics and being clear and decisive. The lack of a significant result in this domain might reflect the need for more in-depth coverage of these aspects in short lectures, complemented by more targeted feedback during training.

Notably, the use of appropriate communication is of utmost importance in the context of psychosocial medicine. Physicians need to use communication to find access to patients, who can be reluctant to disclose their symptoms due to fearing consequences, shame or lack of insight [46]. By improving their communication patterns, international students could also have gained better access to patients. Even though there is a certain difficulty in simulating the entire complexity of patients with mental disorders with SPs, communication training with SPs has shown to improve clinical and communication skills in psychiatric settings [47]. Our study further supports these findings while adding a special focus to psychosomatic disorders and international students as participants. By having more exposure to the subject of psychosomatics, international students might have a better understanding of how to implement a diagnostic approach focused on comprehending psychosocial factors [16].

Furthermore, the controlled statistical analysis in supplement C showed differences in communication patterns between ‘depression’ and ‘PTSD’ cases. As these differences remained stable across study years and thus, across multiple assessment interviews, this finding suggests that communication patterns associated with the underlying mental disorder might vary substantially. For example, the ‘depression’ case was associated with significantly fewer instances of talking over and talk-turn switches in comparison to the ‘PTSD’ case, indicating a fundamentally different communication pattern between both mental disorders. These findings may have important implications for future research. Studies should carefully account for differences between standardized cases by either statistically controlling such differences or, as done in this study, addressing them through randomization. Such differences between standardized cases may influence the interpretation of intervention effects and possibly overshadow improvements in communication skills.

As previously mentioned, the method of NLP in medical education has been developed as a tool to evaluate students’ performance and improve medical educators’ feedback [24, 48, 49]. Comparatively, this study has expanded the application of NLP by defining NLP communication parameters and objectively analyzing international students’ changes in communication style after training. However, as this is the first study to conduct an NLP-based analysis on international students’ communication in training encounters with SPs, there is no prior evidence on expected NLP communication parameters. Consequently, our hypotheses were derived from related literature on international students’ communication performance rather than NLP-specific findings, which necessitated a degree of inferential reasoning.

Nonetheless, the findings of this study suggest that NLP may be a promising complementary approach for the evaluation of communication skills. Furthermore, the results indicate that NLP could be a feasible assessment tool of communication training for international students. NLP communication parameters further provide a novel, additional perspective on interactions between medical students and SPs that may have not been fully captured by the binary checklist domain ‘conversational skills’, as well as global rating domains ‘interview structure’ and ‘verbal expression’. While traditional expert ratings remain essential for assessing subjective aspects of relationship building, such as empathy, responsiveness to verbal and non-verbal cues, mirroring of patients’ emotions, and non-verbal communication, NLP-based analyses might provide more objective insights into communication aspects, including conversation structure, verbal expression, word usage, speaking pace, and communication patterns. Both approaches may therefore complement and enrich one another, allowing for a more comprehensive, detailed and objective analysis of international students’ communication. Combining traditional ratings with NLP-based analyses may allow for a more detailed identification of specific difficulties in communication and relationship building among international students. This combined approach could support medical educators in further developing communication training programs, for example, by incorporating additional teaching units or practice sessions focusing on aspects such as word usage, speech techniques, or conversation structure. Furthermore, NLP communication parameters may enrich feedback processes by providing more objective, precise and potentially more tangible indicators of communication behavior for international students, complementing traditional expert-based evaluations [49]. For example, an expert may subjectively evaluate a student’s communication as more patient-centered following communication training. However, NLP-based analyses may simultaneously reveal persistent communication patterns, such as an excessively high number of questions, that may not have been fully recognized during expert evaluation alone. Such findings could provide additional indications regarding specific communication behaviors that may still require targeted training and feedback.

However, future studies are required to establish substantial guidelines for medical educators on how to apply the NLP method in the evaluation of communication training. These studies should aim to further define NLP communication parameters and establish frameworks for their interpretation in patient-centered contexts, as well as for their application in feedback for medical students. Furthermore, the NLP method could be applied to assess several different medical communication settings, such as in high-stress clinical situations and breaking bad news scenarios.

## Limitations

This study lacks a control group, which could have further supported the results that the improvement in communication skills is an effect of the training seminar. Due to the absence of a control group, alternative explanations for the observed positive results, such as reduced anxiety during post-assessment as a result from increased familiarity with the simulated setting, cannot be ruled out. However, this study establishes the feasibility of NLP communication parameters as an evaluation tool of international students’ communication skills, thus enabling an assessment of the effectiveness of communication training for international students. This lays the foundation for future controlled study designs.

Moreover, by switching the cases between pre- and post-assessments (‘depression’ or ‘PTSD’), both increased familiarity with the cases following training and possible differences in case difficulty may have influenced international students’ performance in post-assessment.

Another limitation concerns the moderate IRR observed for some domains of the binary and global rating instruments. Although several measures were implemented to promote a consistent interpretation of rating items and reduce rater drift, as described in supplement B, variability between raters may still have influenced the results. There remains potential for improvement in future studies. Additional strategies, such as extended rater training, the use of a broader range of example videos with varying interview quality, joint rating sessions, and more frequent calibration meetings, may further improve rating consistency.

Another limitation of this study lies in the lack of accounting for possible linguistic nuances from international medical students, who come from different cultural backgrounds. In the case of fillers, Böttcher and Zellers have previously shown that different languages make use of distinct preferred fillers [50]. Filler usage may also vary within the same language according to gender and bilingualism [50]. Therefore, it is possible that this study may not have accounted for certain filler expressions that are inherent to the different native languages from the study participants.

Furthermore, this study focused on the short-term improvement of international students’ communication parameters. Thus, a long-term outcome on the impacts of communication training on international students’ communication skills is missing. It is possible that international students need multiple communication training sessions to have a sustainable, positive long-term outcome.

Lastly, as indicated by the post hoc power analysis, this study may have been insufficiently powered to reliably detect small effect sizes, suggesting that the effect size assumed in the a priori power analysis was likely overestimated. Given the pilot nature of the study, reliable estimates for expected effect sizes were not available at the time of study design. In this manner, a correction for multiple testing, such as Bonferroni correction, was not applied to the results of this study. Such corrections would have required a larger sample size, which was not feasible within the study’s timeframe and resources.

## Conclusion

To our knowledge, this is the first study to perform an NLP based analysis of the impacts of communication training on international students’ communication skills in psychosocial medicine. This study assessed the objective improvement of international students’ communication skills with NLP after attending a three-day peer-led training seminar. After training, international students might have listened to patients more, had a better interview structure and had a more adequate, confident use of verbal expression. Thus, international students’ communication style might have become, in part, more patient-centered. Furthermore, the NLP method has suggested to be a viable tool to analyze communication patterns in a doctor-patient-relationship and assess the effectiveness of communication training for international students. Taking into consideration all the findings of this study, we believe that a broader implementation of communication training for international medical students across various medical subjects, evaluated with NLP, could be an effective strategy to enhance international students’ communication skills.

## Supplementary Information

Supplementary Material 1.

## Data Availability

Our seminar materials, data frame with rating results, rating instruments, transcription rules, and R markdowns with statistical analysis codes are publicly available under the DOI https://doi.org/10.11588/DATA/2ETDYP.

## Abbreviations

BGR: Berliner Global Rating Scale
HeiTiMed: Heidelberg’s Tutorial for International Medical Students
IRR: Inter-rater reliability
NLP: Natural Language Processing
OSCE: Objective Structured Clinical Examination
PTSD: Post-Traumatic Stress Disorder
SP: Standardized patient
