# Supplementary material for: Improving international medical students’ communication skills in psychosomatic care through a communication training seminar: a natural language processing-based analysis
Source: BMC Med Educ. 2026 Jul 11;26:1155. doi: 10.1186/s12909-026-09890-5 (PMC13371449; doi:10.1186/s12909-026-09890-5)
Supplement: Supplementary file 1 — Supplementary Material 1. [file 12909_2026_9890_MOESM1_ESM.docx]

**Supplements A, B and C to the research article**

**‘Improving international medical Students’ communication skills in psychosomatic care through a communication training seminar: a Natural Language Processing based analysis’**

**Supplement A**

Supplement A provides more detailed information regarding the technical setup in pre- and post-assessment.

**Technical setup**

For pre- and post-assessments, two rooms were prepared with an iPad and a tripod. The iPad was positioned on the tripod in a way that the upper body of both SP and student were in frame. SP and student were seated facing each other. The recorded image was taken from the side. During a diagnostic interview, a tutor was responsible for filming and keeping track of time, as interviews were not allowed to exceed ten minutes.

**Supplement B**

Supplement B provides more detailed information on rater training and rating process.

**Rater training**

From the pool of 78 videos, three videos were chosen to be commented on, based on the items of the rating instruments. The comments were edited into the videos. The chosen videos portrayed a weak, a good, and a very good interview example. The objective of the commented videos was to establish a rating standard for raters, ideally ensuring a good inter-rater reliability. After watching the commented videos, three videos were randomly chosen from the video-pool. All raters watched and rated the randomly chosen videos alone, using both rating instruments as previously instructed. Raters were instructed to complete the binary checklist while viewing the video recording. However, points in domains ‘relationship building’ and ‘conversational skills’ were supposed to be given only in the last two minutes of the video. Global rating was performed directly after finishing the video and the rating of the binary checklist. Raters were also instructed to take notes about their impressions while watching the videos, so that a later discussion could be substantially supported. After rating each video, raters compared and discussed their results. The aim of the rater training was to familiarize raters with the handling of rating instruments, to clear any questions about the rating process and to achieve a common understanding between raters.

**Rating process**

For the rating of diagnostic interviews, three HeiTiMed tutors were recruited as raters (the first, fourth and fifth authors of the manuscript). Before starting the rating process, raters participated in a rater training. All ratings were done on written paper. The 78 videos were randomly divided in 4 video blocks, with around 20 videos each. At the beginning of each video block, all raters were provided 20 copies of the binary checklist and 20 copies of the global rating scale. The order in which videos were rated was randomly determined for each rater, minimizing sequencing effects. After finishing the rating of a video block, raters had a meeting. Rating results were not modified throughout the meeting and/or after it. In the meeting, three videos from the previously rated block were randomly selected and the respective rating results were compared. If rating results deviated strongly between raters, the video was rewatched and raters’ different perspectives were discussed. Hereby, we aimed to avoid a strong rater drift in future ratings and to clear questions that rose through the rating process.

**Supplement C**

In supplement C, we conduct a statistical analysis of possible differences between the student groups, which were randomly built based on which case (‘depression’ or ‘PTSD’) international students were assigned in pre-assessment.

During the review of the manuscript, a reviewer brought up to our attention that the ‘depression’ and ‘PTSD’ cases used in pre- and post-assessments may have different difficulties. Different case difficulties could then have had a reflection in our results, possibly influencing our positive results of an improvement in communication skills. If there were to be differences in case difficulty, we assumed that these differences would have been reflected on international students’ use of NLP communication parameters.

Firstly, it is important to mention that to avoid such differences between groups, all students were randomly assigned a pre-assessment case (either ‘depression’ or ‘PTSD’). These cases are standard cases, which were previously used in OSCEs at the University of Heidelberg. Therefore, we expected the cases to have a similar level of difficulty. International students who received ‘depression’ as their pre-assessment case were assigned ‘PTSD’ for post-assessment, while those who received ‘PTSD’ in pre-assessment were assigned ‘depression’ as their post-assessment case.

Nonetheless, we decided to conduct a controlled statistical analysis regarding the difference between both student groups and thus, possible difficulty differences between cases. With this additional analysis, we hope to be able to answer the reviewer’s question more substantially.

**Methods**

As mentioned above, we randomly separated international students into two groups, by randomly assigning them a pre-assessment case (either ‘depression’ or ‘PTSD’):

Group 1: pre-assessment ‘depression’, post-assessment ‘PTSD’.

Group 2: pre-assessment ‘PTSD’, post-assessment ‘depression’.

First, we calculated the means for both groups for each NLP communication parameter in pre- and post-assessment. Then, we filtered our data frames for pre-assessment. With these ‘pre-dataframes’, we calculated two-way ANOVAs for each NLP communication parameter, thus, assessing international students’ baseline in pre-assessment for both groups. For these ANOVAs, we used group and speakers as between factors.

To control the results of the two-way ANOVAs for the groups, we calculated with our raw dataframes three-way ANOVAs with group and speaker as between factors and session as the within factor. By doing so, we aimed to analyze differences in the use of NLP communication parameters between both groups in both pre- and post-assessments.

The interested reader is referred to the DOI <https://doi.org/10.11588/DATA/2ETDYP>. There, we uploaded our analysis code in detail.

**Results of two-way ANOVAs for NLP communication parameters in pre-assessment**

Table 1 depicts two-way ANOVA results, whereas Table 2 depicts the means of groups for each NLP communication parameter in pre- and post-assessments. The two-way ANOVA results show that there are significant differences between both groups for some of the NLP communication parameters in pre-assessment. The significant differences can be seen for the NLP communication parameters of talk-turn, talk-turn-length and talking over.

- Talk-turn: There are significant more talk-turn switches in Group 2 than in Group 1 in pre-assessment.

- Talk-turn-length: The talk-turn-length in Group 1 is significant longer in comparison to Group 2 in pre-assessment.

- Talking over: Group 2 talks significantly more over patients in comparison to Group 1 in pre-assessment.

In the use of the fillers ‘um’ and ‘mhm’, questions and interruptions, two-way ANOVA results show no significant differences between both groups.

**Table 1.** Two-way ANOVA results for NLP communication parameters in pre-assessment

| **NLP communication parameters** | **Effect** | DF1 | DF2 | F | p | η^2^ |
| --- | --- | --- | --- | --- | --- | --- |
| **Talk-turn** |  |  |  |  |  |  |
|  | Group | 1 | 78 | 20.50 | <0.01 | 0.21 |
|  | Speaker | 1 | 78 | 0.01 | 0.94 | <0.01 |
|  | Group : speaker | 1 | 78 | <0.01 | 0.98 | <0.01 |
| **Talk-turn-length** |  |  |  |  |  |  |
|  | Group | 1 | 78 | 8.10 | <0.01 | 0.10 |
|  | Speaker | 1 | 78 | 12.58 | <0.01 | 0.14 |
|  | Group : speaker | 1 | 78 | 0.07 | 0.79 | <0.01 |
| **Filler ‘um’** |  |  |  |  |  |  |
|  | Group | 1 | 73 | 0.73 | 0.40 | 0.01 |
|  | Speaker | 1 | 73 | 31.25 | <0.01 | 0.30 |
|  | Group : speaker | 1 | 73 | 2.46 | 0.121 | 0.03 |
| **Filler ‘mhm’** |  |  |  |  |  |  |
|  | Group | 1 | 67 | 3.10 | 0.08 | 0.04 |
|  | Speaker | 1 | 67 | 28.81 | <0.01 | 0.30 |
|  | Group : speaker | 1 | 67 | 1.48 | 0.23 | 0.02 |
| **Questions** |  |  |  |  |  |  |
|  | Group | 1 | 78 | 2.15 | 0.15 | 0.03 |
|  | Speaker | 1 | 78 | 231.54 | <0.01 | 0.75 |
|  | Group : speaker | 1 | 78 | 1.02 | 0.32 | 0.01 |
| **Interruptions** |  |  |  |  |  |  |
|  | Group | 1 | 78 | 1.95 | 0.17 | 0.02 |
|  | Speaker | 1 | 78 | <0.01 | 0.99 | <0.01 |
|  | Group: speaker | 1 | 78 | 0.38 | 0.54 | 0.01 |
| **Talking over** |  |  |  |  |  |  |
|  | Group | 1 | 78 | 22.98 | <0.01 | 0.23 |
|  | Speaker | 1 | 78 | 5.57 | 0.02 | 0.07 |
|  | Group : speaker | 1 | 78 | 2.43 | 0.12 | 0.03 |

**Table 2.** Mean $\pm$ standard deviation results for NLP communication parameters in pre- and post-assessments

| **NLP communication parameters** | **Group** | **Assessment** | **Mean** $\boldsymbol{\pm}$ **SD** | |
| --- | --- | --- | --- | --- |
|  |  |  | Student | Patient |
| **Talk-turn** |  |  |  |  |
|  | 1 | Pre | 47.70 $\pm$ 34.74 | 48.20 $\pm$ 34.60 |
|  |  | Post | 50.43 $\pm$ 39.33 | 51.03 $\pm$ 39.12 |
|  | 2 | Pre | 69.79 $\pm$ 48.34 | 69.95 $\pm$ 48.27 |
|  |  | Post | 45.55 $\pm$ 29.98 | 45.73 $\pm$ 29.49 |
| **Talk-turn-length** |  |  |  |  |
|  | 1 | Pre | 11.78 $\pm$ 15.27 | 15.07 $\pm$ 18.31 |
|  |  | Post | 10.48 $\pm$ 12.87 | 16.98 $\pm$ 25.38 |
|  | 2 | Pre | 8.23 $\pm$ 10.49 | 12.26 $\pm$ 15.70 |
|  |  | Post | 11.37 $\pm$ 16.27 | 15.21 $\pm$ 19.10 |
| **Filler ‘um’** |  |  |  |  |
|  | 1 | Pre | 0.08 $\pm$ 0.06 | 0.01 $\pm$ 0.01 |
|  |  | Post | 0.07 $\pm$ 0.04 | 0.02 $\pm$ 0.01 |
|  | 2 | Pre | 0.06 $\pm$ 0.06 | 0.02 $\pm$ 0.02 |
|  |  | Post | 0.06 $\pm$ 0.06 | 0.01 $\pm$ 0.01 |
| **Filler ‘mhm’** |  |  |  |  |
|  | 1 | Pre | 0.03 $\pm$ 0.02 | 0.01 $\pm$ 0.01 |
|  |  | Post | 0.03 $\pm$ 0.03 | 0.01 $\pm$ 0.01 |
|  | 2 | Pre | 0.04 $\pm$ 0.03 | 0.01 $\pm$ 0.01 |
|  |  | Post | 0.03 $\pm$ 0.02 | 0.005 $\pm$ 0.004 |
| **Questions** |  |  |  |  |
|  | 1 | Pre | 26.22 $\pm$ 5.95 | 4.39 $\pm$ 4.45 |
|  |  | Post | 18.67 $\pm$ 5.65 | 3.19 $\pm$ 2.27 |
|  | 2 | Pre | 22.89 $\pm$ 9.21 | 3.78 $\pm$ 3.37 |
|  |  | Post | 23.13 $\pm$ 5.81 | 6.63 $\pm$ 4.05 |
| **Interruptions** |  |  |  |  |
|  | 1 | Pre | 2.74 $\pm$ 3.58 | 3.26 $\pm$ 4.27 |
|  |  | Post | 2.67 $\pm$ 3.06 | 2.19 $\pm$ 2.79 |
|  | 2 | Pre | 4.50 $\pm$ 3.58 | 3.94 $\pm$ 4.22 |
|  |  | Post | 1.56 $\pm$ 1.59 | 2.00 $\pm$ 2.40 |
| **Talking over** |  |  |  |  |
|  | 1 | Pre | 6.35 $\pm$ 5.10 | 4.87 $\pm$ 6.20 |
|  |  | Post | 9.81 $\pm$ 8.78 | 7.43 $\pm$ 8.30 |
|  | 2 | Pre | 18.10 $\pm$ 12.97 | 10.83 $\pm$ 7.95 |
|  |  | Post | 7.25 $\pm$ 7.00 | 3.38 $\pm$ 2.68 |

**Results of three-way ANOVAs for NLP communication parameters**

Table 3 depicts three-way ANOVA results for NLP communication parameters. In the following, we report these results for each NLP communication parameter in detail.

- Filler ‘um’: There are no significant differences between both groups. There are significant differences between speakers, as international students use the filler ‘um’ more frequently than SPs. There are significant differences in the use of the filler ‘um’ between pre- and post-assessments. The means show that both groups reduced the frequency with which they used the filler ‘um’ in post-assessment. The biggest difference can be observed in Group 1. These results are aligned with our previous statistical analysis that international students reduce the frequency with which they used the filler ‘um’.

- Filler ‘mhm’: Significant differences in the use of the filler ‘mhm’ can only be observed between speakers, as international students frequently use this filler to signal active listening. These results are aligned with our previous uncontrolled statistical analysis that international students did not show a significant difference in the use of the filler ‘mhm’ between pre- and post-assessments.

- Talk-turn: There are significant differences between groups. More talk-turn switches between speakers were observed in Group 2 in comparison to Group 1. There are no significant differences between speakers. There are significant differences in the number of talk-turns between pre- and post-assessment. There is a significant interaction between group and session, indicating that changes over time differed between the groups. This interaction effect is highlighted by the means, as Group 1 showed a slight increase, whereas Group 2 exhibited a decrease in the number of talk-turn switches from pre- to post-assessment. Notably, the decrease observed in Group 2 was larger than the increase in Group 1, resulting in an overall reduction in talk-turns, as we have initially reported in the manuscript. It is important to notice that Group 2, by having a larger number of talk-turns, does not necessarily speak more in pre-assessment; it only means that there are more talk-turn switches between international students and patients.

- Talk-turn-length: There are significant differences between groups, as the talk-turn-length from students in Group 1 is significantly longer than from students in Group 2 in pre-assessment. The significant difference between speakers is explained by SPs having longer talk-turn-lengths in comparison to international students. The talk-turn-length from patients has increased significantly in both groups in post-assessment. The talk-turn-length from students in Group 1 has slightly decreased, while the talk-turn-length increased in Group 2, resulting in an overall increase of the talk-turn-length for international students.

- Questions: There are no significant differences between groups. Significant differences between speakers are observed because of the nature of the diagnostic interview – international students ask most of the questions. The significant interaction effect between group and session indicates that changes over time differed between groups. While students in Group 1 reduced the number of questions in post-assessment, Group 2 showed a slight increase. Nonetheless, this results in the overall reduction in the number of questions in post-assessment. The interaction effect between speaker and session indicates that international students reduced significantly the number of questions over time.

- Interruptions: There are no significant differences between groups and speakers. There are significant differences in the number of interruptions between pre- and post-assessments. International student in both groups interrupt patients less in post-assessment.

- Talking over: There are significant differences between speakers, as international students talk more over patients in both pre- and post-assessments. There are significant differences between groups, as students in Group 2 talked more over patients in comparison to students in Group 1 in pre-assessment. However, students in Group 2 strongly reduced their talking over patients in post-assessment, while Group 1 showed an increase in talking over patients. This finding explains the significant differences in talking over between pre- and post-assessments, as well as the significant interaction effects between session and group. Nonetheless, a decrease in talking over patients was observed overall.

**Table 3.** Three-way ANOVA results for NLP communication parameters

| **NLP communication parameters** | **Effect** | DF1 | DF2 | F | p | η^2^ |
| --- | --- | --- | --- | --- | --- | --- |
| **Talk-turn** |  |  |  |  |  |  |
|  | Group | 1 | 60 | 10.89 | <0.01 | 0.11 |
|  | Speaker | 1 | 60 | 0.01 | 0.94 | <0.01 |
|  | Session | 1 | 60 | 14.64 | <0.01 | 0.08 |
|  | Group : speaker | 1 | 60 | <0.01 | 0.10 | <0.01 |
|  | Group : session | 1 | 60 | 13.76 | <0.01 | 0.07 |
|  | Speaker : session | 1 | 60 | <0.01 | 0.98 | <0.01 |
|  | Group : speaker : session | 1 | 60 | <0.01 | 0.99 | <0.01 |
| **Talk-turn-length** |  |  |  |  |  |  |
|  | Group | 1 | 60 | 6.23 | 0.02 | 0.07 |
|  | Speaker | 1 | 60 | 18.14 | <0.01 | 0.17 |
|  | Session | 1 | 60 | 6.64 | 0.01 | 0.03 |
|  | Group : speaker | 1 | 60 | 1.14 | 0.29 | 0.01 |
|  | Group : session | 1 | 60 | 0.16 | 0.70 | <0.01 |
|  | Speaker : session | 1 | 60 | 1.54 | 0.22 | 0.01 |
|  | Group : speaker : session | 1 | 60 | 1.91 | 0.17 | 0.01 |
| **Filler ‘um’** |  |  |  |  |  |  |
|  | Group | 1 | 55 | 1.28 | 0.26 | 0.02 |
|  | Speaker | 1 | 55 | 28.48 | <0.01 | 0.32 |
|  | Session | 1 | 55 | 5.95 | 0.02 | 0.01 |
|  | Group : speaker | 1 | 55 | 0.75 | 0.39 | 0.01 |
|  | Group : session | 1 | 55 | 0.20 | 0.66 | <0.01 |
|  | Speaker : session | 1 | 55 | 3.97 | 0.05 | 0.01 |
|  | Group : speaker : session | 1 | 55 | 6.83 | 0.01 | 0.01 |
| **Filler ‘mhm’** |  |  |  |  |  |  |
|  | Group | 1 | 47 | 0.09 | 0.76 | <0.01 |
|  | Speaker | 1 | 47 | 23.06 | <0.01 | 0.24 |
|  | Session | 1 | 47 | 0.26 | 0.61 | <0.01 |
|  | Group : speaker | 1 | 47 | 1.38 | 0.25 | 0.02 |
|  | Group : session | 1 | 47 | 2.98 | 0.09 | 0.02 |
|  | Speaker : session | 1 | 47 | 0.05 | 0.82 | <0.01 |
|  | Group : speaker : session | 1 | 47 | 0.12 | 0.74 | <0.01 |
| **Questions** |  |  |  |  |  |  |
|  | Group | 1 | 60 | 1.41 | 0.24 | 0.01 |
|  | Speaker | 1 | 60 | 281.43 | <0.01 | 0.74 |
|  | Session | 1 | 60 | 0.72 | 0.40 | 0.01 |
|  | Group : speaker | 1 | 60 | 0.38 | 0.54 | <0.01 |
|  | Group : session | 1 | 60 | 16.18 | <0.01 | 0.10 |
|  | Speaker : session | 1 | 60 | 4.19 | 0.05 | 0.03 |
|  | Group : speaker : session | 1 | 60 | 0.77 | 0.38 | <0.01 |
| **Interruptions** |  |  |  |  |  |  |
|  | Group | 1 | 60 | 0.31 | 0.58 | <0.01 |
|  | Speaker | 1 | 60 | 0.12 | 0.74 | <0.01 |
|  | Session | 1 | 60 | 12.81 | <0.01 | 0.07 |
|  | Group : speaker | 1 | 60 | 0.01 | 0.93 | <0.01 |
|  | Group : session | 1 | 60 | 3.33 | 0.07 | 0.02 |
|  | Speaker : session | 1 | 60 | 0.07 | 0.79 | <0.01 |
|  | Group : speaker : session | 1 | 60 | 0.55 | 0.46 | <0.01 |
| **Talking over** |  |  |  |  |  |  |
|  | Group | 1 | 60 | 5.05 | 0.03 | 0.05 |
|  | Speaker | 1 | 60 | 6.89 | 0.01 | 0.07 |
|  | Session | 1 | 60 | 9.83 | <0.01 | 0.05 |
|  | Group : speaker | 1 | 60 | 1.68 | 0.20 | 0.02 |
|  | Group : session | 1 | 60 | 33.38 | <0.01 | 0.16 |
|  | Speaker : session | 1 | 60 | 0.32 | 0.58 | <0.01 |
|  | Group : speaker : session | 1 | 60 | 1.58 | 0.21 | <0.01 |

**Discussion**

The three-way ANOVA analysis shows that there are differences between both groups in the use of some NLP communication parameters. Some differences between groups were reflected in both pre- and post-assessments. Nonetheless, the results of the three-way ANOVAs align themselves with the results of our initial uncontrolled statistical analysis, which are reported in the manuscript. This controlled statistical analysis has additionally shown that there are some differences between groups as well as interaction effects with the groups. This suggests that one of the groups exhibits a slightly greater change across assessments, either for patients or international students, or in the interaction between patient, student, group and session.

Furthermore, this controlled analysis showed that differences in the use of NLP communication parameters, especially in the use of talk-turns and talk-turn-length, are strongly associated with the speakers. Studies have shown that depressed patients present a slower speech rate as well as a reduced speech intensity, for example, through longer and more frequent pauses [1]. On the other hand, PTSD patients have shown a higher word count to express complex emotions [2]. Our controlled statistical analysis has shown that PTSD interviews were characterized by having more talk-turns, reduced talk-turn-length and more talking over from both patients and students, in comparison to depression interviews.

Therefore, differences between the groups are not necessarily associated with differences between case difficulty, but rather with differences in communication patterns associated with the mental disorders portrayed in the assessment cases. These differences in communication patterns between mental disorders should be accounted for in future study designs with controlled statistical analysis as well as with randomization, thus, avoiding a possible overshadowing of the improvement of international students’ communication skills.

**References**

1. Cummins N, Scherer S, Krajewski J, Schnieder S, Epps J, Quatieri TF. A review of depression and suicide risk assessment using speech analysis. Speech Commun. 2015; 71:10-49. https://doi.org/10.1016/j.specom.2015.03.004

2. Yu Z, Gu Z, Shen Y, Lu J. The relationship between language features and PTSD symptoms: a systematic review and meta-analysis. Front Psych. 2025; 16:1476978. doi:10.3389/fpsyt.2025.1476978
